# Association of carbamylated high-density lipoprotein with coronary artery disease in type 2 diabetes mellitus: carbamylated high-density lipoprotein of patients promotes monocyte adhesion

**DOI:** 10.1186/s12967-020-02623-2

**Published:** 2020-12-03

**Authors:** Zhongli Chen, Song Ding, Yan Ping Wang, Liang Chen, Jing Yan Mao, Ying Yang, Jia Teng Sun, Ke Yang

**Affiliations:** 1 Department of Vascular & Cardiology, Ruijin Hospital, Shanghai Jiao Tong University School of Medicine, 197 Ruijin Road II, Shanghai, 200025 People’s Republic of China; 2 Department of Cardiology, Ren Ji Hospital, Shanghai Jiao Tong University School of Medicine, 160 Pujian Road, Shanghai, 200027 People’s Republic of China; 3 Department of Cardiac Surgery, State Key Laboratory of Cardiovascular Disease, Fuwai Hospital, National Center for Cardiovascular Diseases, Chinese Academy of Medical Sciences and Peking Union Medical College, Beijing, China; 4 Department of Endocrinology, The Second People’s Hospital of Yunnan Province, Kunming, 650021 Yunnan China

**Keywords:** Carbamylation, Carbamyl-lysine,coronary artery disease, High-density lipoprotein, Monocyte adhesion, Type 2 diabetes mellitus

## Abstract

**Background:**

Increasing evidence showed that carbamylated lipoprotein accelerated atherosclerosis. However, whether such modification of high-density lipoprotein (HDL) particles alters in type 2 diabetes mellitus (T2DM) patients and facilitates vascular complications remains unclear. We aimed to investigate the alteration of the carbamylation in HDL among T2DM patients and clarify its potential role in atherogenesis.

**Methods:**

A total of 148 consecutive T2DM patients undergoning angiography and 40 age- and gender-matched control subjects were included. HDL was isolated from plasma samples, and the concentration of HDL carbamyl-lysine (HDL-CBL) was measured. Furthermore, the HDL from subjects and in-vitro carbamylated HDL (C-HDL) was incubated with endothelial cells and monocyte to endothelial cell adhesion. Adhesion molecule expression and signaling pathway were detected.

**Results:**

Compared with the control group, the HDL-CBL level was remarkably increased in T2DM patients (6.13 ± 1.94 vs 12.00 ± 4.06 (ng/mg), *P* < 0.001). Of note, HDL-CBL demonstrated a more significant increase in T2DM patients with coronary artery disease (CAD) (n = 102) than those without CAD (n = 46) (12.75 ± 3.82 vs. 10.35 ± 4.11(ng/mg), *P* = 0.001). Multivariate logistic regression analysis demonstrated that higher HDL-CBL level was independently associated with a higher prevalence of CAD in diabetic patients after adjusting for established cofounders (adjusted odds ratio 1.174, 95% confidence Interval 1.045–1.319, p = 0.017). HDL from diabetic patients with CAD enhanced greater monocyte adhesion than that from the non-CAD or the control group (*P* < 0.001). Such pro-atherogenic capacity of diabetic HDL positively correlated with HDL-CBL level. Furthermore, in-vitro incubation of carbamylated HDL (C-HDL) with endothelial promoted monocyte to endothelial cell adhesion, induced upregulation of cell adhesion molecules expression, and activated NF-κB/p65 signaling in endothelial cells. Inhibiting carbamylation of HDL or NF-κB activation attenuated the monocyte to endothelial cell adhesion and cell surface adhesion molecules expression.

**Conclusions:**

Our study identified elevated carbamylation modification of HDL from T2DM patients, especially in those with concomitant CAD. We also evidenced that C-HDL enhanced monocyte to endothelial cell adhesion, indicating a potential pro-atherogenic role of C-HDL in atherosclerosis among T2DM patients.

*Trial registration* https://register.clinicaltrials.gov, NCT04390711 Registered on 14 May 2020; Retrospectively registered

## Background

High-density lipoprotein (HDL) is involved in various athero-protective processes, including reverse cholesterol transport [1], inhibition of lipid oxidation and inflammatory cytokine secretion, endothelial repair, and anti-apoptotic function, which all contribute to the regression of plague burden [2]. However, new evidence suggests that HDL can undergo profound alteration in composition under certain pathological conditions such as diabetes [3, 4]. In a hyperglycemic environment, several post-translational modifications of protein take place, with oxidative modification, one of the most well-established harmful mechanisms rendering HDL dysfunctional [5]. Oxidized or glycoxidized HDL is generally considered pro-atherogenic and is associated with reduced cholesterol efflux capacity and impaired anti-inflammatory activity, eventually contributing to the initiation and progression of coronary artery disease (CAD) [6–9]. Interestingly, recent studies have shown that oxidative stress and chronic inflammation—both implicated in the process of diabetes—can contribute to an irreversible post-translational modification called carbamylation [10]. Protein carbamylation is produced by interactions between cyanate and free amino groups, which preferentially occur on the e-NH2 of lysine residues and generate carbamyl-lysine (CBL), a characteristic carbamylation-derived product [11]. Cyanate, which is formed by myeloperoxidase-catalyzed oxidation of thiocyanate or by urea dissociation, is increased at inflammatory sites and in chronic kidney disease [12, 13]. Thus, carbamylation can reflect the burden of enhanced inflammation, oxidative stress, and renal impairment, and can serve as a biomarker of certain pathological conditions [13, 14]. Several clinical studies have demonstrated positive associations between cardiovascular risk, mortality, and serum carbamylation-derived product levels in the general population and particularly in patients with kidney failure [15, 16]. The underlying mechanism that links carbamylation to high CAD risk is the positive charge neutralization of lysine and compositional change of targeted proteins. As a result, carbamylated proteins harbor atherogenic properties and can promote organ dysfunction [17]. Increasing evidence shows that carbamylated lipoprotein plays a pivotal role in atherosclerosis [18]. Our previous study, along with others, showed that carbamylated modification of HDL impairs its endothelial repair properties as well as reverses cholesterol transport activities [19, 20]. Notably, most studies concerning carbamylation have been performed under a kidney disease background. Whether HDL particles in patients with type 2 diabetes mellitus (T2DM) show enhanced carbamylation and whether this enhancement is associated with vascular complications remain unknown.

Thus, in the present study, we tested the hypothesis that HDL experiences carbamylation in T2DM patients, who generally exhibit elevated oxidative stress and inflammation. Furthermore, we investigated the pro-atherogenic effects of carbamylated HDL. To this end, we incubated in-vitro C-HDL particles with endothelial cells to evaluate their role in the induction of monocyte adhesion, cell surface adhesion molecule expression, and related pathway activation.

## Methods

### Study subjects

Between September 2018 and March 2019, a total of 242 consecutive T2DM patients who underwent coronary angiography/intervention in Ruijin hospital due to typical angina symptom and/or electrocardiographic ST-T wave changes were enrolled in this study. Diagnosis of T2DM was made according to the criteria of the American Diabetes Association [21]. All patients were tested by angiography, with CAD diagnosed if luminal diameter narrowing was estimated visually at ≥ 50% in a major epicardial coronary artery. The SYNTAX score of CAD participants was calculated by 2 interventional cardiologists using the online calculator (http://www.syntaxscore.com).

**Fig. 1:**
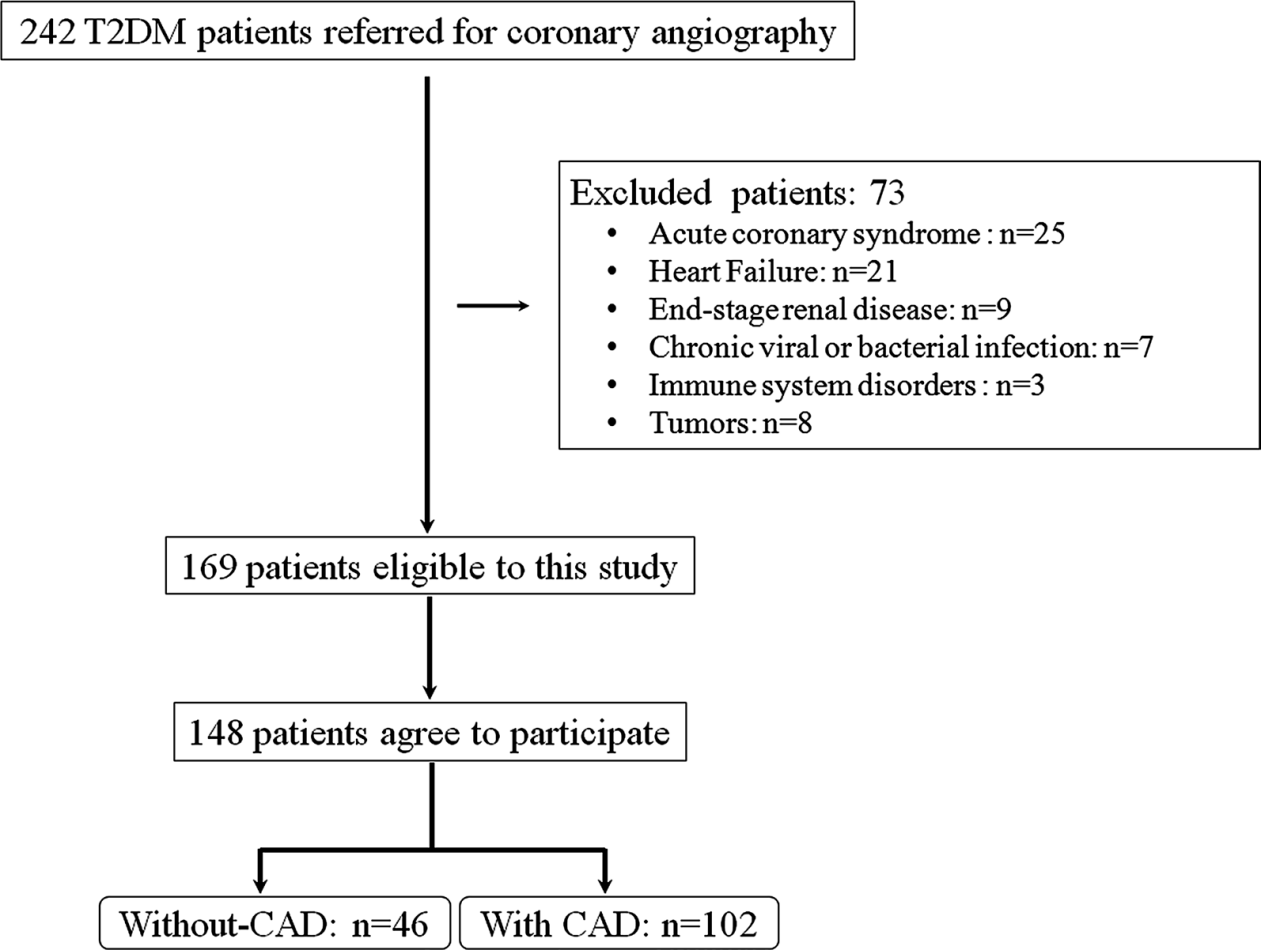
Flowchart of patients enrollment

Patients with end-stage renal disease (n = 9), acute coronary syndrome (n = 25), heart failure (n = 21), chronic viral or bacterial infection (n = 7),tumors(n = 8),or immune system disorders(n = 3) were excluded from this study to avoid confounding influence. We also excluded patients who declined to enter the study (n = 21) (Fig. 1). We also included 40 age- and gender-matched subjects from the physical examination center of our hospital to serve as the control group. These subjects were free of diabetic and vascular risk factors based on history and laboratory examination. Subjects with present abnormal blood pressure, serum fasting glucose, lipid profile, and renal function were also excluded.

This study protocol was approved by the Institutional Review Board of Ruijin Hospital, Shanghai Jiao Tong University School of Medicine. Each subject provided written informed consent and all clinical investigations were conducted in accordance with the ethical standards of the Helsinki Declaration.

### Biochemical measurements in patients

All blood samples were taken on the day of cardiac catheterization after overnight fasting. Serum level of glucose, glycated hemoglobin A1c (HbA1c), creatinine, blood urea nitrogen (BUN), and lipid profiles were detected with standard laboratory techniques. The glomerular filtration rate (GFR) was estimated with the Modification of Diet in Renal Disease (MDRD) formula.

### Lipoprotein analysis

Fresh plasma HDL (density 1.063 to 1.21 g/mL) was isolated by standard density-gradient ultracentrifugation in a Beckman Optima Ultracentrifuge with a fixed angle rotor and then excessively dialyzed at 4 °C against 0.15 mol/l NaCl and 0.01% ethylenediaminetetraacetic acid (EDTA) at pH 7.0 four times over 48 h. The concentration of isolated HDL was measured by SDS-PAGE followed by Coomassie blue staining, according to apolipoproteinA-1 (apoA-1) content. Both carbamylation and glycation of HDL were separately determined using human enzyme-linked immunosorbent assay (ELISA) kits. Carbamylation of HDL was expressed as HDL carbamyl-lysine (CBL) ng per milligram of protein, whereas glycation was expressed as carboxyethyl lysine (CEL) ng per milligram of protein.

### In-vitro Carbamylation of HDL

Fresh fasting plasma from 10 healthy individuals was pooled. HDL was isolated and then incubated (20 mg/mg HDL) with potassium cyanate (KOCN) at 37 °C for 4h [19, 20], followed by excessive dialysis as mentioned above. The cyanate concentration used in our study followed the method describled in the previous research [22]^.^. Control HDL was prepared under the same conditions without the use of KOCN. To inhibit carbamylation of HDL, the pooled HDL were incubated with the inhibitor glycylglycine (MCE:HY-D0889) at 250 mmol/l as previously described in the presence of KOCN at 37 °C for 4 h [23, 24].

### Cell culture and adhesion assay

In brief, human umbilical vein endothelial cells (HUVECs) were cultured in EC medium (ScienCell cat 1001) supplemented with 5% FBS, 1% penicillin/streptomycin solution and EC growth supplements. HUVECs between 2 to 4 passages were seeded at a concentration of 3 × 10 [4] cells/well onto 24-well plates, and, after reaching confluence, they were starved overnight in 1% FBS ECM culture medium and incubated in ECM basic culture medium containing either isolated HDL (100ug/mL) or sodium chloride (control) at 37 °C for 6 h prior to the adhesion assay. Supernatants were then collected and measured by ELISA to determine vascular cell adhesion molecule-1 (VCAM-1) and intercellular adhesion molecule-1 (ICAM-1) level. For NF-κB inhibition, Dehydroxymethylepoxyquinomicin ((-)-DHMEQ) (MCE 287194-40-5), a potent and selective inhibitor of NF-κB were used to pretreat the HUVECs. HUVECs were pretreated with 5 mg/mL DHMEQ for 6 h as described in previous publications [25], and then were exposed to 100 µg/mL HDL for 6 h and subsequently subjected to adhesion assay. After the stimulation, the culture media were switched to adhesion cell suspension (RPMI-1640Gibco11875093) containing calcein-labeledTHP-1monocyte cell line at 40,000 cells per well. After 1 h, non-adherent monocytes were removed by being washing with phosphate-buffered saline (PBS). Adherent monocytes were counted on three randomly selected fields at 4× magnification using a fluorescence microscope.

### NF-κB/p65 immunofluorescence

Immunofluorescence staining was used to detect NF-κB/p65 subunit nuclear translocation. Endothelial cells were cultured on glass coverslips. After stimulation, the cells were fixed with 4% paraformaldehyde for 10 min, followed by permeabilization in 0.2% Triton X-100 for 20 min, and then blocked with 5% BSA for 1 h. Subsequently, the glass coverslips were incubated with phospho-NF-κB antibody (1:200) for 12 h, followed by secondary antibody (FITC-conjugated donkey anti-rabbit IgG, 1:1 000) incubation at room temperature for 1 h. Afterwards, cells were stained with 5 ng/mL 4′6-diamidino-2-phenylindole (DAPI, Beyotime, Shanghai, China) and coverslips were fixed on the glass slides. Immunofluorescence signals were visualized using a laser confocal microscope (Zeiss LSM 710 system, Oberkochen, Germany), with images then analyzed using ZEN 2.3 (Zeiss).

### Western blotting

The expression level of ICAM-1, VCAM-1, total p65, and phosphorylated p65 were analyzed by western blotting. Briefly, endothelial cells were lysed using RIPA buffer (ThermoFisher, Waltham, MA, USA). Total proteins of equal concentrations were loaded and electrophoretically separated on 10% SDS/PAGE. The proteins were then blotted onto nitrocellulose membranes. After blotting, the membranes were incubated with primary antibody (1:1 000) and then with corresponding horseradish peroxidase-conjugated secondary antibody (1:5000). Afterwards, proteins were visualized using a chemiluminescence detection system (Millipore, MA, USA). Images were captured using a Tanon-5500 chemiluminescent imaging system (Tanon Science and Technology Co., Ltd., Shanghai, China) and quantification of band intensity was performed using ImageJ software (Bio-Rad, Hercules, CA, USA). Each experiment was done in triplicate.

### Flow cytometry

In brief, single-cell suspensions of endothelial cells were prepared after Carbamylated-HDL/healthy HDL incubation. Afterwards, endothelial cells were stained with direct FITC-ICAM-1 fluorescent antibody and PE-Cyanine7-VCAM-1 antibody. Samples were acquired on CytoFLEX S flow cytometer (Beckman Coulter) and analyzed with CyExpert 2.0 (Beckman Coulter).

#### Real-time polymerase chain reactions (PCR)

Total RNA was extracted from HCVEC using Trizol reagent. Reverse transcription was performed using 5 μg total RNA. Real-time PCR was performed within a StepOne System (Applied BioSystems, CA, USA)system, using the Power SYBR Green PCR Master Mix (Applied BioSystems, CA, USA) for relative mRNA quantification. Primers are listed in Additional file 1 Table S1.

### Reagents and antibodies

Primary and secondary antibodies against proteins ICAM-1, VCAM-1, p65, p-p65, and β-actin were purchased from Cell Signaling Technology (MA, USA). The CBL and OxiSelect™ Nε-(carboxyethyl) lysine ELISA kits were purchased from Cell Biolabs (USA). The myeloperoxidase (MPO) ELISA kit was from Mercodia (Uppsala, Sweden). The ICAM-1 and VCAM-1ELISA kits were obtained from Antigenix (American Inc, NY, USA). All antibodies and ELISA kits were applied according to the instructions provided by the manufacturers. Potassium bromide, KOCN, and all other reagents were from Sigma-Aldrich (St. Louis, MO, USA) (Additional file 1: Table S1).

### Statistical analysis

Data were presented as means ± standard deviation for continuous variables that were normally distributed, or as median ± interquartile range (IQR) for the non-normally distributed ones as percentages for categorical variables. Continuous variables were compared by unpaired Student’s *t*-tests or one-way analysis of variance(ANOVA)if they were normally distributed; otherwise, Mann–Whitney U-tests or Kruskal–Wallis tests were performed. Chi-square tests were applied for comparing categorical variables. Pearson or Spearman bivariate correlational analyses were conducted to investigate the correlation between HDL carbamyl-lysine (HDL-CBL) level and other variables as appropriate. Multiple linear regression was used to examine the associations between HDL-CBL and the following factors: age, HbA1c, fasting glucose, BUN, GFR, MPO and hsCRP. Multivariate logistic regression analyses were performed to determine the independent association between HDL-CBL level and CAD prevalence in T2DM patients while adjusting for other confounders including age, systolic blood pressure, hsCRP, HbA1c, fasting glucose, LDL-C concentration, smoking, GFR and HDL-CEL level. Statistical analysis was performed with SPSS (IBM SPSS 23.0, SPSS Inc). Graphical analyses were conducted in GraphPad Prism 8.0 (GraphPad Software, La Jolla, CA). Results were considered statistically significant when a two-sided P value was less than 0.05.

## Results

### Clinical features of T2DM patients and healthy controls

Overall, we included 148 T2DM patients, including 102 patients with CAD and 46 without CAD. Demographic and clinical characteristics of the study cohort are summarized in Table 1. As expected, patients with T2DM had higher glucose level and HbA1c level than the healthy controls. The prevalence of other conventional CAD-related risk factors, such as blood pressure and lipid metrics, was dysregulated in the T2DM groups. Among the 148 T2DM patients, a higher level of fasting blood glucose, HbA1c, creatinine, and MPO level were observed in T2DM patients with CAD, compared with those without CAD. Subjects with CAD were more frequently on statin treatment. There were no significant differences in lipid profile, blood pressure, insulin therapy, or hypoglycemic medication use between the two groups.

**Table 1:** Baseline characteristics and biochemical assessments

	Healthy control (n = 40)	T2DM patients	*P*-value	T2DM without CAD (n = 46)	T2DM with CAD (n = 102)	*P*-value
Age, years	61.99 ± 4.88	62.89 ± 11.67	NS	60.54 ± 14.4	65.0 ± 8.9	NS
Male/female	25/15	96/52	NS	29/17	67/35	NS
BMI kg/m^2^	24.56 ± 4.21	25.62 ± 3.43	NS	25.4 ± 4.1	25.7 ± 3.0	NS
SBP mmHg	124.20 ± 13.79	136.86 ± 14.84	< 0.001	135.8 ± 12.3	137.2 ± 15.9	NS
DBP mmHg	70.02 ± 8.82	75.58 ± 9.58	< 0.001	74.7 ± 8.8	76.0 ± 10.0	NS
Smoking (n, %)	12 (30%)	45 (30.4%)	NS	15 (32.6%)	30 (29.4%)	NS
Alcohol (n, %)	11 (27.5%)	46 (31.1%)	NS	15 (33.3%)	31 (30.4%)	NS
Triglycerides (mmol/L)	1.46 ± 1.05	1.77 ± 1.15	< 0.001	1.78 ± 1.28	1.75 ± 1.09	NS
Total cholesterol (mmol/L)	4.35 ± 0.73	4.28 ± 0.92	NS	4.17 ± 0.64	4.32 ± 1.03	NS
HDL cholesterol (mmol/L)	1.54 ± 0.339	1.05 ± 0.23	< 0.001	0.97 ± 0.26	1.05 ± 0.23	NS
LDL cholesterol (mmol/L)	2.35 ± 0.59	2.47 ± 0.83	NS	2.44 ± 0.50	2.48 ± 0.94	NS
Fasting glucose (mmol/L)	5.35 ± 0.34	7.75 ± 1.82	< 0.001	7.29 ± 1.70	7.98 ± 1.84	0.033
HbA1c (%)	5.57 ± 0.21	7.74 ± 1.27	< 0.001	7.43 ± 1.19	8.03 ± 1.24	0.006
Creatinine (µmol/L)	77.31 ± 8.81	76.70 ± 37.4	NS	67.63 ± 20.22	78.16 ± 21.13	0.005
Blood urea nitrogen (mmol/L)	6.21 ± 0.89	6.41 ± 2.34	NS	5.90 ± 1.78	6.55 ± 1.96	NS
GFR (mL/min/1.73 m^2^)	86.18 ± 7.54	88.70 ± 22.30	NS	97.86 ± 19.60	84.31 ± 17.45	< 0.001
HDL-CEL level (ng/mg)	25.41 ± 10.35	36.63 ± 18.06	< 0.001	32.92 ± 17.81	38.31 ± 18.01	NS
HDL-CBL level (ng/mg)	6.13 ± 1.94	12.00 ± 4.06	< 0.001	10.35 ± 4.11	12.75 ± 3.82	0.001
hs-CRP level (mg/L)				0.67 (0.10–1.20)	0.83 (0.35–2.51)	NS
MPO level (μg/L)				164.6 ± 28.4	179.0 ± 31.69	0.009
Medical treatment						
Crossover treatment				9 (19.6%)	23 (22.5%)	NS
Insulin therapy				15 (32.6%)	37 (36.2%)	NS
Metformin				20 (43.4%)	50 (49.0%)	NS
Sulphonyl urea				13 (28.3%)	29 (28.4%)	NS
a-Glucosidase				24 (52.2%)	45 (44.1%)	NS
Statin				25 (54.3%)	83 (81.4%)	0.01
Anti-hypertensive drugs				28 (60.8%)	65 (63.7%)	NS

*BMI* body mass index, *GFR* glomerular filtration rate, *CAD* coronary artery disease, *T2DM* type 2 diabetes mellitus, *HbA1c* glycated hemoglobin A1c, *SBP* systolic blood pressure, *DBP* diastolic blood pressure, *CBL* carbamyl-lysine, *CEL* carboxyethyl lysine, *hs-CRP* high-sensitive C response protein, *MPO* myeloperoxidase, *NS* not significant

### Carbamylation level of HDL in T2DM patients

To assess *in-vivo* HDL carbamylation, we measured the CBL level in HDL isolated from the T2DM and control groups. Results showed that HDL carbamylation was increased in T2DM patients (HDL-CBL: 12.00 ± 4.06 ng/mg vs 6.13 ± 1.94 ng/mg, *P* < 0.001) (Table 1).We further tested the potential factors that might influence HDL-CBL level using simple and multiple linear regression analyses. Linear correlation showed that HDL-CBL concentration was positively correlated with MPO level (Pearson’s r = 0.406, *P* < 0.001) and hsCRP concentration (Spearman’s r = 0.198, p = 0.029), but was negatively associated with GFR (Pearson’s r = -0.186, *P* = 0.024). Of note, in multiple linear regression analysis, the MPO level was the only independent variable that remained significantly associated with the level of HDL-CBL (β = 0.397, *P* < 0.001) (Table 2).

**Table 2:** Factors associated with CBL levels in diabetic HDL

Variable	Univariate linear correlation		Multiple linear regression	
	*r*	*P*-value	*β*	*P*-value
Age (years)	0.073	NS	− 0.002	NS
HbA1c (%)	0.073	NS	0.074	NS
Fasting glucose (mmol/L)	0.021	NS	− 0.034	NS
Blood urea nitrogen (mmol/L)	0.094	NS	− 0.016	NS
GFR (mL/min/1.73 m)	− 0.186	0.024	− 0.122	NS
MPO (μg/L)	0.406	< 0.001	0.397	< 0.001
hsCRP (mg/L)	0.198	0.029	0.082	NS

*CBL* carbamyl-lysine, *MPO* myeloperoxidase, *HbA1c* glycated hemoglobin A1c, *GFR* glomerular filtration rate, *hsCRP* high-sensitive C response protein, *NS* not significant, *r* univariate correlation coefficients between HDL-CBL level and other variables, *β* multiple linear regression standardized coefficients

### Associations between HDL-CBL level, HDL-CEL level and CAD prevalence/severity

We next evaluated the relationship between HDL-CBL level and CAD prevalence in T2DM patients. We found that HDL-CBL level was higher in T2DM patients with CAD than those without (12.75 ± 3.82 ng/mg vs 10.35 ± 4.11 ng/mg, P = 0.001). As glycation is another important determinant of diabetic HDL dysfunction, we detected level of CEL (a major glycation product) in T2DM patients. HDL-CEL was increased in T2DM patients compared with healthy subjects (25.41 ± 10.35 ng/mg vs 36.63 ± 18.06 ng/mg, *P* < 0.001), however, we failed to detect an increase in the CAD subgroup compared to the subgroup without CAD. Multivariate logistic regression analysis revealed that HDL-CBL level (adjusted OR 1.176 95% CI: 1.030–1.343, p = 0.017), rather than HDL-CEL level, were independently associated with CAD prevalence, after adjusting for traditional cardiovascular risk factors including age, systolic blood pressure, hsCRP, HbA1c, fasting glucose, LDL-C concentration, smoking, GFR and HDL-CEL level (Table 3). Moreover, CAD patients were further divided into multi-vessel disease group and one-vessel disease group according to the number of stenotic coronary vessels and then the SYNTAX score was calculated. We found that HDL-CBL level was higher in the multi-vessel disease group compared with that of the one-vessel disease group (13.41 ± 3.32 ng/mg vs 11.64 ± 4.35 ng/mg, P < 0.05). We also observed a significant positive correlation between HDL-CBL level and SYNTAX score (r = 0.390, P < 0.001), indicating that a higher level of HDL-CBL might be associated with CAD severity of theseT2DM patients. (Additional file 2: Figure S1).

**Table 3:** Logistic regression analysis of CAD incidence in T2DM patients

Variable	OR (95% CI)	*P*-value
Age (years)	0.988 (0.939–1.040)	NS
SBP (mmHg)	1.004 (0.972–1.038)	NS
HbA1c %	1.501 (0.971–2.321)	NS
Fasting glucose (mmol/L)	1.098 (0.816–1.478)	NS
Smoking	1.625 (0.600–4.402)	NS
LDL cholesterol (mmol/L)	0.778 (0.425–1.423)	NS
GFR (mL/min/1.73 m^2^)	0.951 (0.918–0.987)	0.007
HDL-CBL level (ng/mg)	1.174 (1.045–1.319)	0.017
HDL-CEL level (ng/mg)	1.012 (0.986–1.038)	NS
hs-CRP (mg/L)	1.074 (0.894–1.291)	NS

*CI* confidence interval, *OR* odds ratio, HbA1c glycated hemoglobin A1c, *BMI* body mass index, *CAD* coronary artery disease, *T2DM* type 2 diabetes mellitus, *CBL* carbamyl-lysine, *CEL* carboxyethyl lysine, *hsCRP* high sensitive C response protein, *SBP* systolic blood pressure, *GFR* glomerular filtration rate, *NS* not significant

### Association between HDL carbamylation level and pro-inflammatory ability

**Fig. 2:**
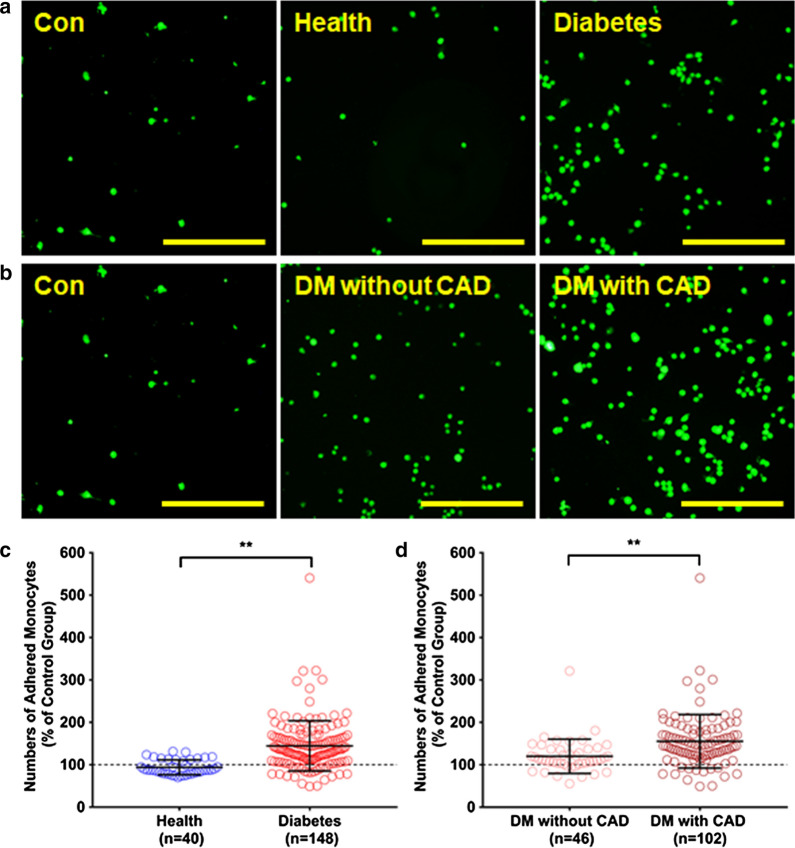
Monocyte adhesion in response to human HDL. **a** HUVECs were treated with HDL from healthy controls and from diabetic patients with or without CAD for 6 h and then co-cultured with monocytes for 1 h. Control group was treated with sodium chloride. **b** Adherent monocytes were counted on three randomly selected fields at 4 × magnification and typical photographs were taken (**P < 0.01)

**Fig. 3:**
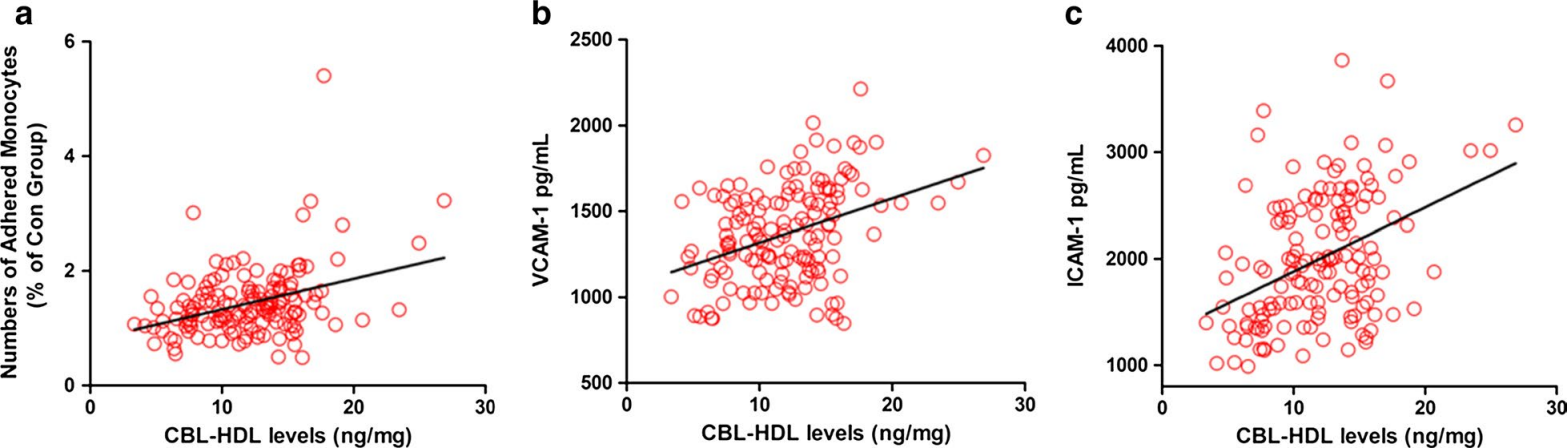
Association between HDL-CBL level and monocyte adhesion. **a** Correlation plot of HDL-CBL level and monocyte adhesion number. **b** Correlation plot of HDL-CBL level and secreted VCAM-1 level. **c** Correlation plot of HDL-CBL level and and secreted ICAM-1 level

To further evaluate the potential pro-atherogenic properties of HDL carbamylation, HDL-induced monocyte adhesion was performed as monocyte is a crucial event to endothelial adhesion in plaque formation. HUVECs were treated with HDL from T2DM patients and healthy controls and then co-cultured with monocytes. As shown in Fig. 2, the diabetic HDL substantially increased the number of monocytes to endothelial cells adhesion compared to the control HDL, with the CAD subjects-derived HDL promoting the most significant monocyte adhesion. Correlation analysis indicated that HDL-CBL level correlated with adhered monocyte numbers positively (Pearson coefficient r = 0.364; *P* < 0.001). Since the cell adhesion molecules play a vital role in intercellular communication in the process of cell adhesion, we also measured the concentration of secreted cell adhesion molecules in HUVEC supernatant after HDL-C stimulation. Consistently, we found that HDL-CBL level were also positively associated with VCAM-1 (Pearson coefficient r = 0.374; *P* < 0.001) and ICAM-1 (Pearson coefficient r = 0.407; *P* < 0.001) levels in the supernatant (Fig. 3).

### C-HDL in-vitro induced monocyte adhesion and related pathway activation

**Fig. 4:**
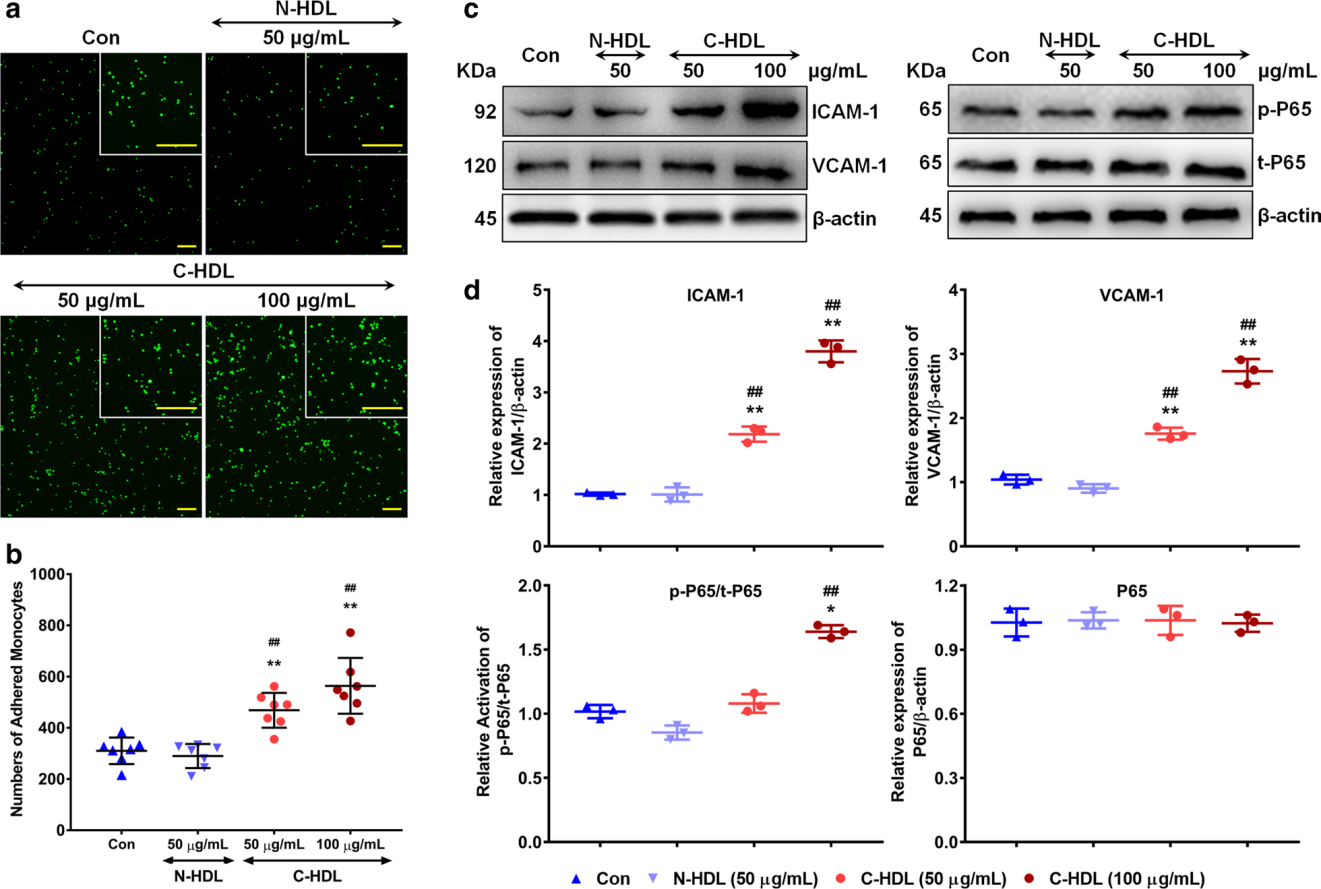
Effects of carbamylated HDL (C-HDL) on monocyte adhesion and related pathway activation. **a**, **b** HUVECs were treated with HDL from healthy controls and C-HDL for 6 h, then co-cultured with monocytes for 1 h. Control group was treated with sodium chloride. Adherent monocytes were counted on 7 randomly selected fields at 4 × magnification, with typical photographs taken. **c,** **d** Representative immunoblots and quantitative analysis of protein level of ICAM-1, VCAM-1, p65, and phosphorylation of p65. (**P < 0.01 compared with control, ## compared with normal HDL)

To investigate the effect of CBL enrichment in HDL on the induction of monocyte adhesion and pathway activation, we performed HDL carbamylated modification with cyanate *in-vitro*. Results showed that C-HDL treatment significantly promoted monocytes to HUVEC adhesion in a dose-dependent manner. By contrast, normal-HDL-treated HUVECs did not show increased monocyte adhesion (Fig. 4a, b). Consistently, C-HDL treatment lead to upregulated ICAM-1 and VCAM-1 expression of HUVECs in both protein and mRNA level (Fig. 4c, d, Additional file 3: Figure S2). The upregulation of cell surface adhesion molecule expression induced by C-HDL was also testified by flow cytometry method (Additional file 4: Figure S3). These results suggest that C-HDL might induce monocyte to endothelial cell adhesion and enhance the cell adhesion molecules expression in endothelial cell.

**Fig. 5:**
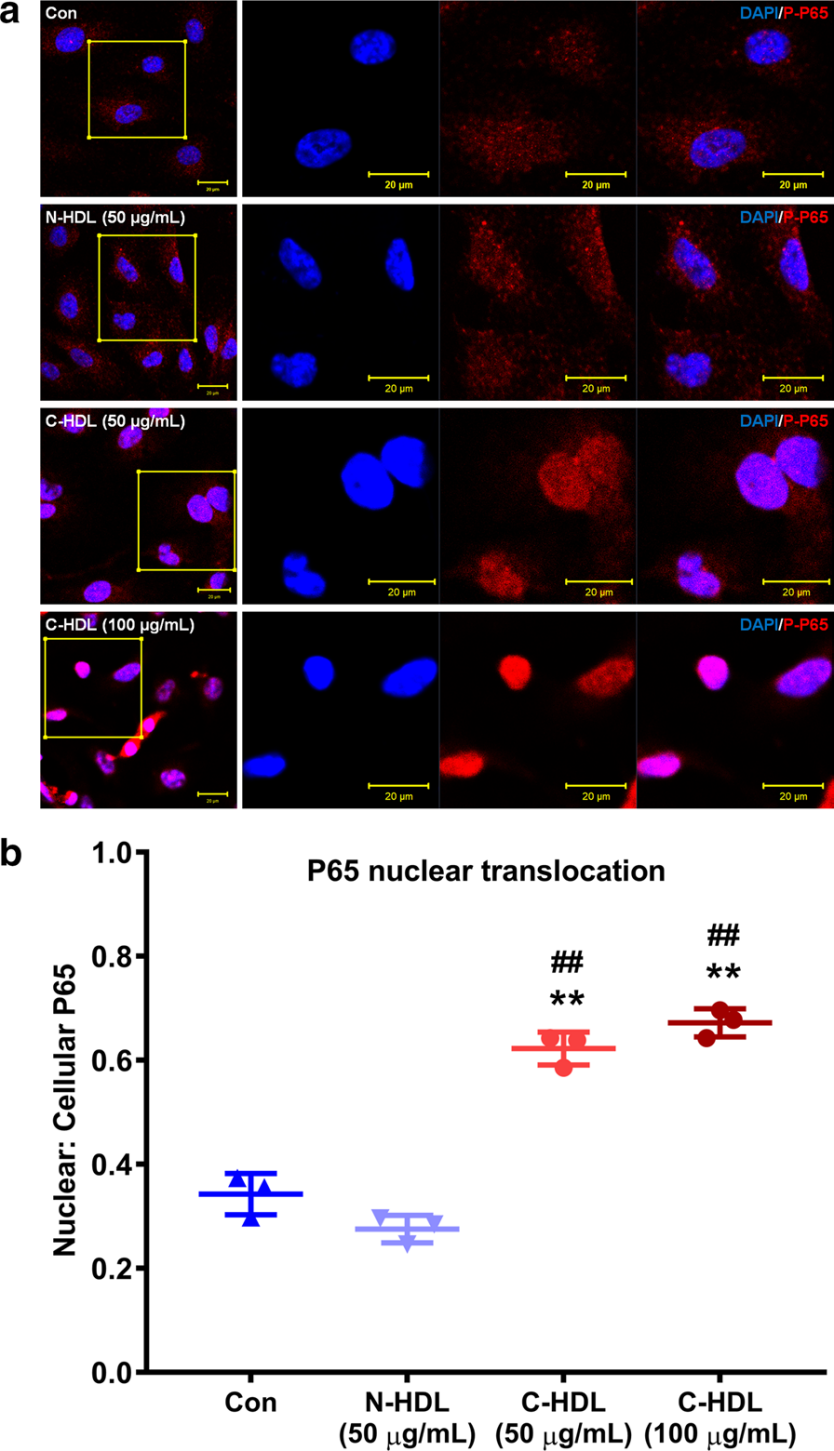
Nuclear translocation of nuclear transcription factor kappa-B (NF-κB). **a** Nuclear translocation of nuclear transcription factor kappa-B (NF-κB) was detected by immunofluorescence staining in 3 cell climbing slices in each group. And 3 visual fields of each slice were chosen randomly. **b** Quantitative analysis of nuclear translocation of NF-κB. Results were showed as ratio of nuclear/nuclear + cytoplasmic NF-κB integrated optical density (IOD). Data points represent mean ± standard deviation (n = 3 each group). (**P < 0.01 compared with control, ## compared with normal HDL)

**Fig. 6:**
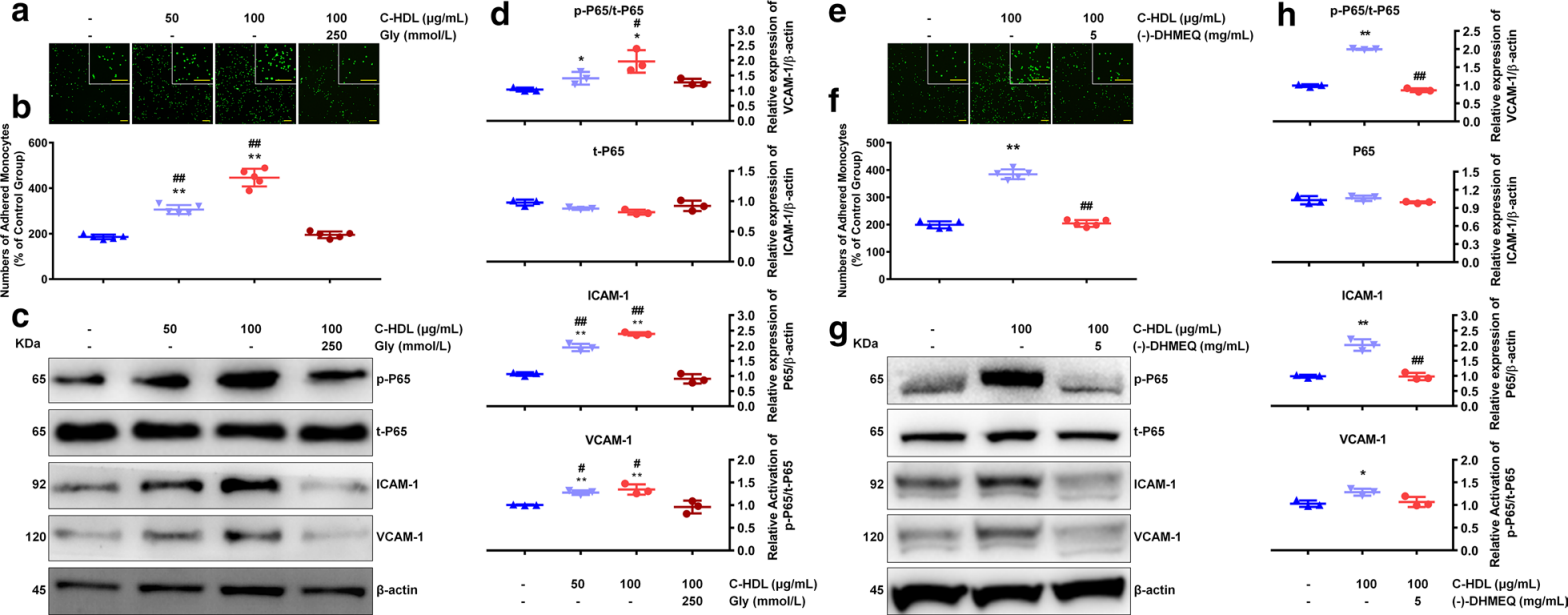
Impact of inhibiting HDL carbamylation or NF-κB activation on monocyte adhesion and adhesion molecules expression. **a**, **b** HUVECs were treated with C-HDL (50 μg/mL), C-HDL (100 μg/mL) glycylglycine-incubated HDL (100 μg/ mL) for 6 h, then co-cultured with monocytes for 1 h. Control group was treated with sodium chloride. Adherent monocytes were counted on 5 randomly selected fields at 4 × magnification, with typical photographs taken. **c**,** d** Representative immunoblots and quantitative analysis of protein level of ICAM-1, VCAM-1, p65, and phosphorylation of p65. (**P < 0.01 compared with control, ^##^P < 0.01 compared with glycylglycine-incubated HDL). **e**, **f** HUVECs with and without pretreatment of 5 mg/mL (-)- DHMEQ were exposed to C-HDL for 6 h (100 μg/ mL), then co-cultured with monocytes for 1 h. Control group was treated with sodium chloride. Adherent monocytes were counted on 5 randomly selected fields at 4 × magnification, with typical photographs taken. **g,** **h** Representative immunoblots and quantitative analysis of protein level of ICAM-1, VCAM-1, p65, and phosphorylation of p65. (**P < 0.01 C-HDL group compared with control group, ^##^P < 0.01, (-)- DHMEQ pretreated group compared with C-HDL group)

Given that NF-κB played an important role in the upregulation of ICAM-1 and VCAM-1 in several disease conditions [26–29], we aimed to detect whether NF-κB/p65 signal contributed to C-HDL-induced monocyte to HUVEC adhesion. We found that under C-HDL treatment, the phosphorylation of p65 in HUVECs was remarkably increased, compared to the untreated or normal-HDL treated HUVECs without influencing total p65 level (Fig. 4c, d). Additionally, C-HDL also significantly promoted p65 subunit translocation to the nucleus in HUVECs (Fig. 5). Furthermore, after inhibiting carbamylation of HDL by glycylglycine, the extend of monocyte to HUVEC adhesion was attenuated, compared with the C-HDL treated groups (Fig. 6a, b). And western blotting analysis showed that after inhibiting carbamylzation of HDL, expression of ICAM-1, VCAM-1 in HUVECs decreased, which was accompanied by reduced phosphorylation of NF-κB/p65 (Fig. 6c, d). In consistency, when inhibiting NF-κB activation through pretreating the HUVECs with the NF-κB inhibitor (-)-DHMEQ, C-HDL failed to induced more monocyte adhesion (Fig. 6e, f). In (-)-DHMEQ pretreated HUVECs, the activation of NF-κB was remarkably suppressed, and expression of ICAM-1, as well as VCAM-1, also decreased significantly even in the presence of C-HDL (Fig. 6g, h), indicating that NF-κB might contribute to the C-HDL induced monocyte adhesion and cell adhesion molecules upregulation.

## Discussion

To the best of our knowledge, this study is the first to report increased C-HDL level in T2DM patients and its association with CAD prevalence and severity. First of all, we found that increased HDL-CBL level in T2DM patients was related to increased oxidative stress rather than renal impairment. Secondly, HDL from diabetic patients with CAD, in contrast to those without, significantly promotes monocyte adhesion. Of note, the pro-inflammatory ability of diabetic HDL was associated with HDL-CBL level. Thirdly, C-HDL increased monocyte-endothelium adhesion and cell adhesion molecule expression via NF-κB/p65 signal activation. These findings suggested that the process of HDL carbamylation might be associated with the high risk of atherosclerotic burden in T2DM patients.

It is well-established that T2DM lead to accelerated atherosclerosis with increased activation of neutrophils and prolonged release of reactive oxygen species (ROS) in diabetic cardiovascular cells [30]. Overproduction of ROS not only participates in various pathogenic signaling pathways but also directly damages protein function by inducing protein oxidation. Carbamylation, formed by protein post-translational modification with cyanate, is a specific form of oxidation and increased in low-density lipoprotein (LDL) particles of T2DM patients [31]. We measured CBL level in HDL isolated from T2DM patients and healthy controls, which showed, for the first time, that HDL-CBL content increased in T2DM patients. More importantly, the level of such modification was positively associated with the presence and severity of concomitant CAD. In addition, elevated HDL-CBL concentration was independently associated with higher CAD prevalence after adjusting for traditional CAD risk factors.

Another important finding of our study is that MPO-mediated protein oxidation may serve as a major pathway for the generation of C-HDL. MPO, the most abundant protein in leukocytes, exerts catalytically active function in atherosclerotic lesions [32]. MPO catalyzes thiocyanate in the presence of hydrogen peroxide and induces cyanate formation at inflammation sites, thus promoting targeted protein carbamylation [33]. Our study also demonstrated that CBL content in the HDL of T2DM patients was positively correlated with MPO concentration, even after adjustment in multiple linear regression, implicating the concurrently elevated concentration of MPO in T2DM patients in our study, which might serve as a driving force for the enhanced carbamylation in such patients. It is also necessary to note that no significant correlation was observed between HbA1c and C-HDL level among T2DM patients, which is in line with the previous study [34]. This might be due to the limited direct participation of HbA1c in lipoprotein carbamylation. Basically, carbamylation is mainly driven by decomposition of urea or MPO mediated protein oxidation and previous publication also revealed that MPO-lowing therapy attenuated the carbamylated-lipoprotein concentration, and the reduction in plasma carbamylated-LDL correlated with changes in MPO rather than HbA1c [31]. Hence, even though poor glycemic control might contribute to enhanced oxidative stress and declined renal function, the lack of association between HbA1c and C-HDL may hint more complicated pathogenesis beyond the simple theoretical linkage.

Hyperglycemic-induced HDL is associated with increased cardiovascular risk, as advanced glycation end products (AGEs) exert conformational change on HDL and hamper its anti-inflammatory ability [35, 36]. Hence, we also detected the level of CEL, a major antigenic AGE, on HDL. Although increased HDL-CEL level was observed in T2DM patients, we failed to detect a positive association between HDL-CEL level and CAD prevalence in T2DM individuals. We speculated that glycation induced a variety of AGEs, and that the CEL level measured in this study did not adequately represent total AGE accumulation in HDL. In addition, many other factors, such as aging, dietary intake, and renal function, can impact AGE concentration [37, 38].

The association between HDL-CBL and CAD increased our interest in exploring the contribution of C-HDL to the process of arthrosclerosis pathophysiology. Isolated HDL particles from all subjects were incubated with HUVECs to test their effect on endothelial adhesiveness and adhesion molecule expression. Accumulating evidence has demonstrated that dysfunctional HDL is not only losing the athero-protective functions [39], but also gaining inflammatory characteristics and can exacerbate the vascular injury [40]. The loss of the anti-atherogenic effects of HDL were frequently observed in diabetic patients [36, 41]. However, the evidence on HDL functional alterations in T2DM patients with and without CAD are scarce. Notably, in the present study, we observed that HDL from T2DM patients with CAD promoted greater monocyte-endothelium adhesion than that from T2DM patients without CAD or healthy controls. Such pro-atherogenic capacity of diabetic HDL also displayed a positive correlation with HDL-CBL content, supporting the notion that HDL carbamylation is involved in the pathogenesis of atherosclerosis. For this purpose, in-vitro C-HDL was incubated for endothelial-monocyte assay to examine the mechanism underlying the pro-inflammatory activity of C-HDL. In fact, C-HDL remarkably enhanced monocyte-endothelium adhesion and increased expression of ICAM-1 and VCAM-1 in HUVECs. And inhibiting carbamylation of HDL attenuated this pro-atherogenic capacity of C-HDL.

The previous study has demonstrated that MPO-catalyzed oxidation of HDL in the vessel wall activates nuclear factor NF-κB and promotes arterial inflammation [42]. NF-κB is one of the most important transcription mediators involved in inflammatory responses and strongly influences monocyte recruitment [43]. We, therefore, tested the hypothesis that exposure of C-HDL to endothelial cells would induce NF-κB activation. Western blotting and immunofluorescence data revealed that C-HDL significantly enhanced the nuclear translocation of NF-κB/p65 as well as its phosphorylation level in HUVECs compared with the normal HDL group and control group. And inhibition of Nf-κB significantly relieved the effects of C-HDL on monocyte adhesion and adhesion molecule expression. These results support the notion that C-HDL participates in inflammatory cytokine expression and monocyte adhesion via activation of the NF-κBsignaling pathways.

Admittedly, this study has several limitations. First of all, as a cross-sectional study without time-to-event information, the cause-effect relationship could not be established with certainty. A large-scale, prospective study is, therefore needed to further assess our results. Secondly, our study lacked proteomic data concerning different carbamylated sites in diabetic HDL, and prospective studies that quantify specific carbamylated residues in circulating HDL might provide a more useful indication of HDL dysfunction and related CAD risk. Finally, the NF-κB signaling pathway might not be the only mediator for C-HDL induced monocyte-to HUVEC adhesion. Further studies are needed for detecting multiple possibilities and the complicated network regulations for the signaling pathway regulation on vascular cells by this new modification of HDL.

## Conclusions

In conclusion, our study identified elevated carbamylation level as a novel feature of HDL from T2DM patients with concomitant CAD. *In-vitro* evidence supported a pro-atherogenic role of carbamylated-HDL in the promotion of monocyte adhesion. Thus, carbamylation of HDL might be an important factor contributing to HDL dysfunction and development of atherosclerotic plaque in T2DM patients. Further studies are required to explore the comprehensive impact of carbamylated HDL in the dynamic process of diabetic cardiovascular disease.

## Supplementary information

**Additional file 1: Table S1.** Reagents and materials.

**Additional file 2: Figure S1.** Association of HDL-CBL levels and CAD severity. A. Difference of HDL-CBL levels between the one-vessel and multi-vessel disease (*P < 0.05). B. Correlation plot of HDL-CBL levels and SYNTAX score.

**Additional file 3: Figure S2.** mRNA fold change of ICAM-1 and VCAM-1 under C-HDL stimulation. A. Fold change of ICAM-1 and B, VCAM-1 at mRNA level among the control, normal-HDL ( 50 μg/mL) treated, C-HDL( 100 μg/mL) treated and C-HDL( 50 μg/mL) treated HUVECs. Data points represent mean ± standard deviation (n = 3 each group). (**P < 0.01 compared with control, ## compared with normal HDL, using one‐way ANOVA followed by Tukey's post hoc test).

**Additional file 4: Figure S3.** Flow cytometry analysis of surface ICAM-1 and VCAM-1 expression. A. HUVECs were treated with normal-HDL, C-HDL( 50 μg/mL) and C-HDL( 100 μg/mL)then analyzed by flow cytometry for surface ICAM-1 and VCAM-1 expression. B. C-HDL induced increased proportion of ICAM-1 + VCAM-1 + endothelial significantly. n = 3 independent experiments; (**P < 0.01 compared with control, ## compared with normal HDL, using one‐way ANOVA followed by Tukey's post hoc test).

## Data Availability

The datasets used and/or analyzed during the current study are available from the corresponding author on reasonable request.

## Abbreviations

T2DM: Type 2 diabetes mellitus
CAD: Coronary artery disease
HDL: High-density lipoprotein
CBL: Carbamyl-lysine
C-HDL: Carbamylated-high-density lipoprotein
HbA1c: Glycated hemoglobin A1c
CEL: Carboxyethyl lysine
MPO: Myeloperoxidase
HUVECs: Human umbilical vein endothelial cells
VCAM-1: Vascular cell adhesion molecule-1
ICAM-1: Intercellular adhesion molecule-1
