# Supplementary figures and images for: Association of carbamylated high-density lipoprotein with coronary artery disease in type 2 diabetes mellitus: carbamylated high-density lipoprotein of patients promotes monocyte adhesion
Source: J Transl Med. 2020 Dec 3;18:460. doi: 10.1186/s12967-020-02623-2 (PMC7713164; doi:10.1186/s12967-020-02623-2)

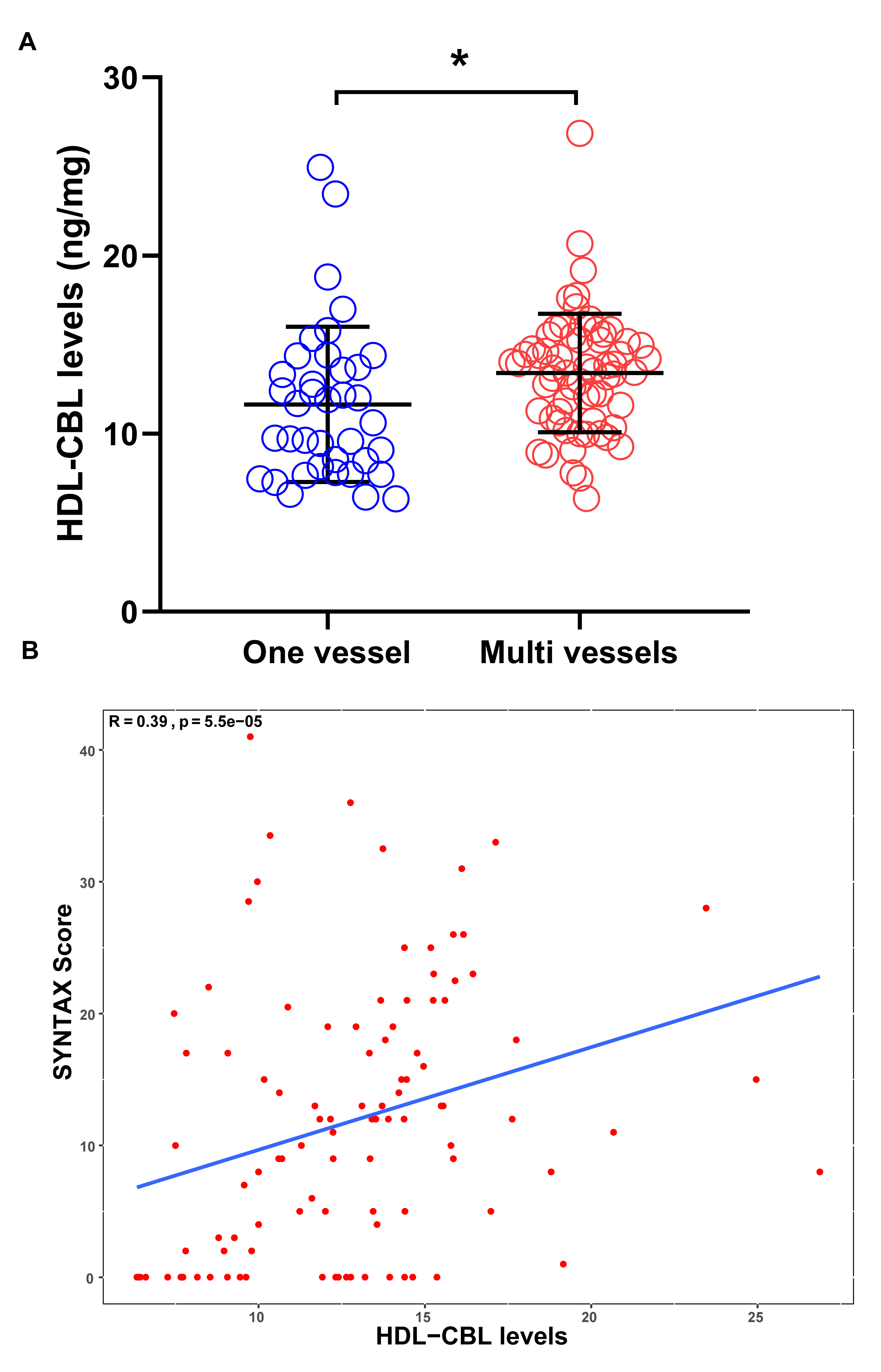

Supplement: Supplementary file 1 — Additional file 1: Table S1. Reagents and materials. [file 12967_2020_2623_MOESM1_ESM.tif]

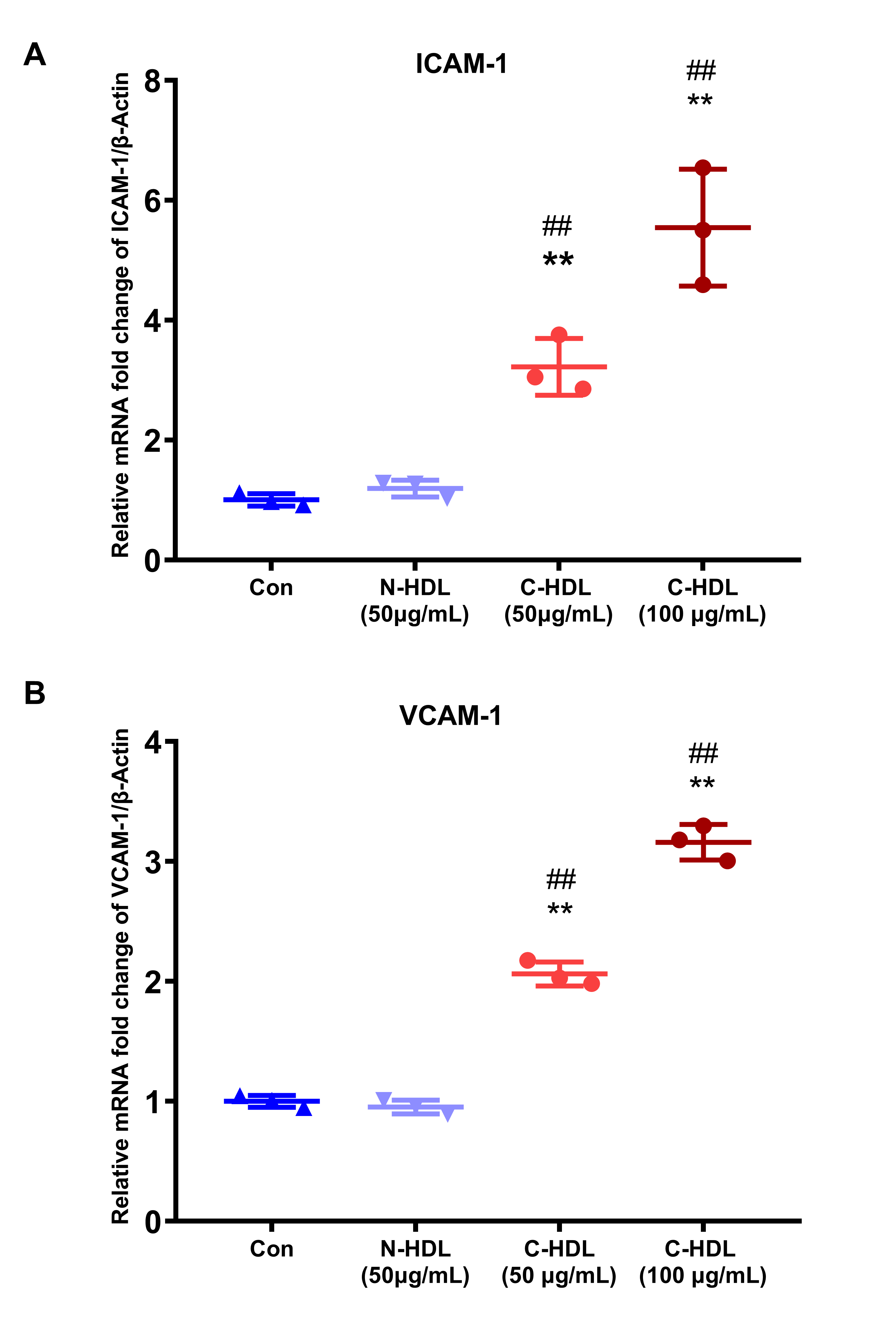

Supplement: Supplementary file 2 — Additional file 2: Figure S1. Association of HDL-CBL levels and CAD severity. A. Difference of HDL-CBL levels between the one-vessel and multi-vessel disease (*P < 0.05). B. Correlation plot of HDL-CBL levels and SYNTAX score. [file 12967_2020_2623_MOESM2_ESM.tif]

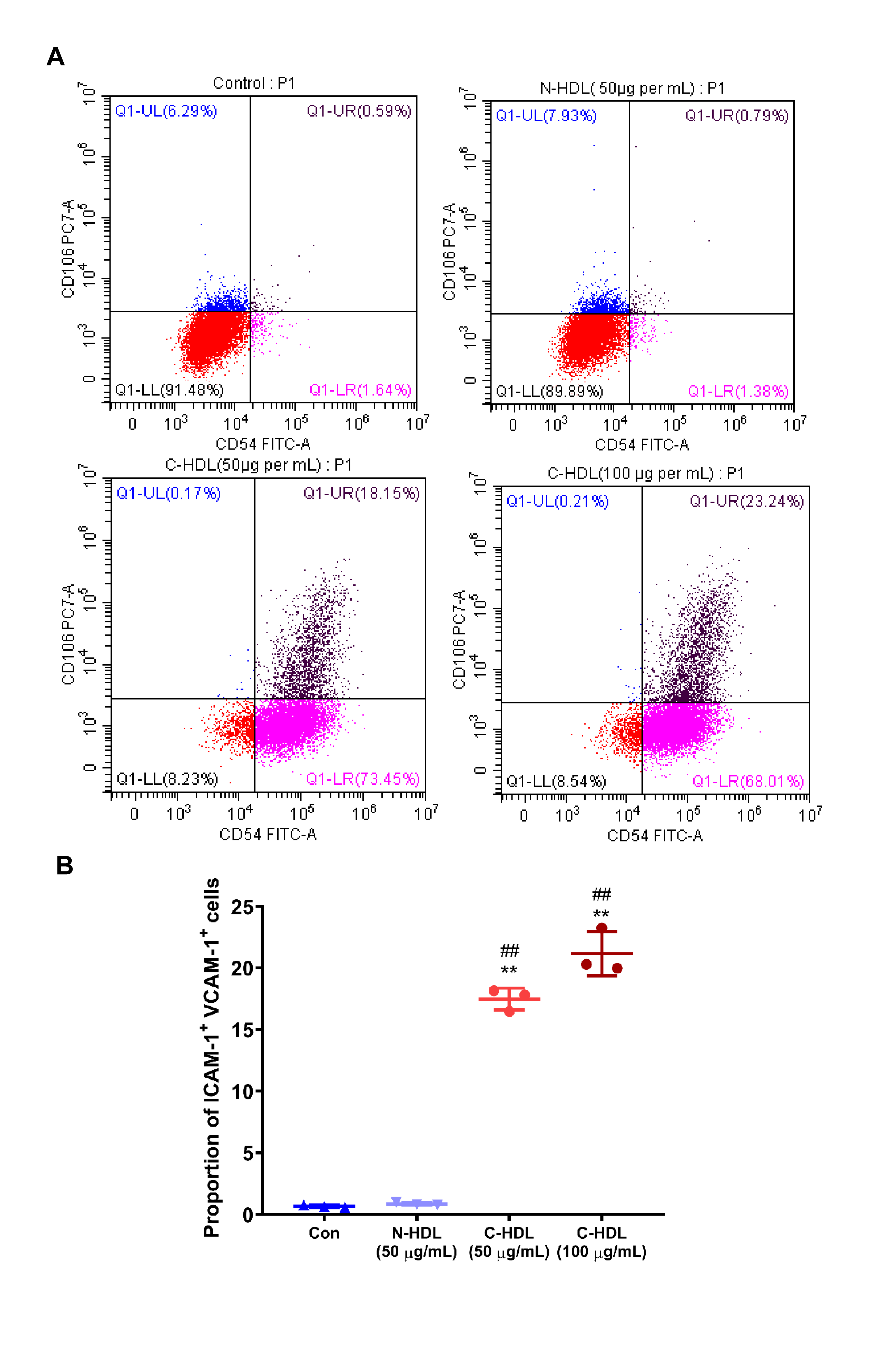

Supplement: Supplementary file 3 — Additional file 3: Figure S2. mRNA fold change of ICAM-1 and VCAM-1 under C-HDL stimulation. A. Fold change of ICAM-1 and B, VCAM-1 at mRNA level among the control, normal-HDL ( 50 μg/mL) treated, C-HDL( 100 μg/mL) treated and C-HDL( 50 μg/mL) treated HUVECs. Data points represent mean ± standard deviation (n = 3 each group). (**P < 0.01 compared with control, ## compared with normal HDL, using one‐way ANOVA followed by Tukey's post hoc test). [file 12967_2020_2623_MOESM3_ESM.tif]
