# Supplementary material for: Association of carbamylated high-density lipoprotein with coronary artery disease in type 2 diabetes mellitus: carbamylated high-density lipoprotein of patients promotes monocyte adhesion
Source: J Transl Med. 2020 Dec 3;18:460. doi: 10.1186/s12967-020-02623-2 (PMC7713164; doi:10.1186/s12967-020-02623-2)
Supplement: Supplementary file 4 — Additional file 4: Figure S3. Flow cytometry analysis of surface ICAM-1 and VCAM-1 expression. A. HUVECs were treated with normal-HDL, C-HDL( 50 μg/mL) and C-HDL( 100 μg/mL)then analyzed by flow cytometry for surface ICAM-1 and VCAM-1 expression. B. C-HDL induced increased proportion of ICAM-1 + VCAM-1 + endothelial significantly. n = 3 independent experiments; (**P < 0.01 compared with control, ## compared with normal HDL, using one‐way ANOVA followed by Tukey's post hoc test). [file 12967_2020_2623_MOESM4_ESM.docx]

**Table S1: Reagents and Materials**

| Name | Company | Catalog |
| --- | --- | --- |
| CD54/ICAM-1 (E3Q9N) XP® Rabbit mAb | **CST** | **67836** |
| VCAM-1 (E1E8X) Rabbit mAb | **CST** | **13662** |
| β-Actin (8H10D10) Mouse mAb | **CST** | **3700** |
| NF-κB p65 (D14E12) XP® Rabbit mAb | **CST** | **8242** |
| Phospho-NF-κB p65 (Ser536) (93H1) Rabbit mAb | **CST** | **3033** |
| CD106 (VCAM-1) Monoclonal Antibody (STA), PE-Cyanine7 | **eBioscience** | **25-1069-42** |
| FITC anti-human CD54 Antibody | **biolegend** | **353108** |
| glycylglycine | **MCE** | **HY-D0889** |
| (-)-DHMEQ | **MCE** | **287194-40-5** |
| HUVEC | **ATCC** | **PCS-100-010** |
| THP-1 | **ATCC** | **TIB-202** |
| Potassium bromide | **Sigma-Aldrich** | **451010** |
| Potassium cyanate | **Sigma-Aldrich** | **215074** |
| Protein Carbamylation Sandwich ELISA | **Cell Biolabs (USA)** | **STA-877** |
| CEL Competitive ELISA | **Cell Biolabs (USA).** | **STA-813** |
| MPO ELISA | **Mercodia (Uppsala, Sweden)** | **10-1176-01** |
| Human ICAM-1 Construction Kit - | **Antigenix (American Inc, NY, USA)** | **RHF717CK** |
| Human VCAM-1 Construction Kit | **(American Inc, NY, USA)** | **RHF506CK** |
| RPMI-1640 | **Gibco** | **11875093** |
| EC Medium | **ScienCell** | **1001** |
| 24-well plates | **CORNING** | **3524** |
| Primers | **Forward Sequence** | **Reverse Sequence** |
| ICAM-1 | CAGAGGTTGAACCCCACAGT | GAGACCTCTGGCTTCGTCAG |
| VCAM-1 | CAGTAAGGCAGGCTGTAAAAGA | TGGAGCTGGTAGACCCTCG |
| Beta-Actin | CGGGAAATCGTGCGTGACAT | AAGGAAGGCTGGAAGAGTGC |
